# The influence of past research on the design of experiments with dissolved organic matter and engineered nanoparticles

**DOI:** 10.1371/journal.pone.0196549

**Published:** 2018-05-07

**Authors:** Nicole Sani-Kast, Patrick Ollivier, Danielle Slomberg, Jérôme Labille, Konrad Hungerbühler, Martin Scheringer

**Affiliations:** 1 Institute for Chemical and Bioengineering, ETH Zürich, Zürich, Switzerland; 2 Bureau de Recherches Géologiques et Minières (BRGM), Orléans, France; 3 Aix-Marseille Université, CNRS, IRD, INRA, Coll France, CEREGE, Aix-en-Provence, France; 4 RECETOX, Masaryk University, Brno, Czech Republic; Iowa State University, UNITED STATES

## Abstract

To assess the environmental fate of engineered nanoparticles (ENPs), it is essential to understand their interactions with dissolved organic matter (DOM). The highly complex nature of the interactions between DOM and ENPs and other particulate matter (PM) requires investigating a wide range of material types under different conditions. However, despite repeated calls for an increased diversity of the DOM and PM studied, researchers increasingly focus on certain subsets of DOM and PM. Considering the discrepancy between the calls for more diversity and the research actually carried out, we hypothesize that materials that were studied more often are more visible in the scientific literature and therefore are more likely to be studied again. To investigate the plausibility of this hypothesis, we developed an agent-based model simulating the material choice in the experiments studying the interaction between DOM and PM between 1990 and 2015. The model reproduces the temporal trends in the choice of materials as well as the main properties of a network that displays the DOM and PM types investigated experimentally. The results, which support the hypothesis of a positive reinforcing material choice, help to explain why calls to increase the diversity of the materials studied are repeatedly made and why recent criticism states that the selection of materials is unbalanced.

## Introduction

Understanding the environmental fate of engineered nanoparticles (ENPs) is a major step towards nanotechnology risk assessment [1, 2]. In the environment, transformation processes such as aggregation and dissolution determine the distribution of ENPs in water, sediment and soil. In water, for example, rapid aggregation results in fast deposition of ENPs to the sediment layer and therefore might limit their mobility, while ENP stabilization enables the particles to travel for longer distances and subsequently contaminate larger areas [3, 4]

Dissolved organic matter (DOM), a material that is ubiquitous in the aquatic environment and comprises diverse compounds such as humic substances and polysaccharides [5], strongly affects ENP aggregation and dissolution rates [6]. The nature of the interaction between DOM and ENPs is not yet fully resolved because it depends on multiple and interrelated factors such as pH, ionic strength and types of ions present, and the chemical properties of both DOM and ENPs [7–9]. Because of this complexity, it is unlikely that the interactions between DOM and ENPs can be fully characterized by using only mechanistic approaches. Consequently, Louie et al. (2016) proposed to establish correlations between experimental conditions and experimental outcomes as an alternative approach to a full mechanistic understanding of DOM-ENP interactions [10].

To obtain insights applicable to a broad range of DOM and ENP types, both from correlations and from mechanistic studies, there is a need to study the interactions between DOM and particulate matter (PM), i.e., ENPs and particles of larger size, for a range of chemically and structurally diverse DOM and ENP types [11]. For this reason, recent reviews have called to diversify the types of DOM and PM studied in the experiments [10, 12]. However, in our previous work we found that there is an overall decrease, rather than an increase, in the diversity of studied DOM-PM combinations [11]. PM types studied most often are titanium dioxide and silver nanoparticles; rarely studied are, e.g., quantum dots (note that PM and ENPs are not distinct categories, but PM includes ENPs). On the DOM side, river humic acids are predominantly investigated, whereas humic substances from peat and seawater are much less often studied. An overview of the DOM and PM types investigated in different years is provided in Figure 3 of Sani-Kast et al. (2017) [11]. Experiments with particles of different size were included here because (i) size is a relevant factor that may affect DOM-PM interactions and (ii) there are also many other relevant factors (particle properties other than size; properties of the DOM; conditions of the aqueous medium) and a sufficiently broad empirical basis that reflects the influences of these factors is needed.

The decreasing material diversity (expressed as the number of materials studied per number of experiments performed) can originate from various reasons such as a research focus on materials of high environmental interest (e.g. ENPs that are toxic and produced in large quantities and DOM from regions near ENP emission sources), preference to study relatively simple and well characterized materials (e.g. uncoated and insoluble ENPs or fully characterized DOM fractions), or other specific interests and funding considerations.

Recently, Maynard and Aitken explicitly called for investigations into the factors that drive researchers to focus on a rather small subset of materials [13]: “For example, the nanotechnology safety research portfolio has become increasingly unbalanced over the past decade, with toxicology research far outstripping exposure-based research, and research into specific materials (silver and titanium dioxide for example) has not always aligned well with likely potency or impact. Understanding how such imbalances emerge, and how to counter them, will provide important insights for future strategic research.” [13]

Here, in response to this call, we aim to understand the dynamics behind the choices of DOM and PM to be studied and discuss possible driving forces that account for the observed imbalance in the choice of materials. Going beyond the empirical findings presented by Sani-Kast et al. (2017) [11], we present an agent-based model (ABM) that simulates the choices of DOM and PM types made by researchers in experiments between 1990 and 2015. This type of model allows us to analyze how the choices made collectively by many agents drive the spectrum of the DOM and PM types investigated (the actions of individual researchers and, in particular, how the pattern of the investigated PM and DOM types emerges from these actions were not addressed by Sani-Kast et al. (2017) [11]).

An important observation from the empirical field is that materials studied frequently in the past are more likely to be re-studied; we call this process “frequency-based material choice”, FBMC. Frequency-based material choice is understood here as a path-dependent and a self-reinforcing process (i.e., the choice of materials at a given time affects the likelihood of their future choice). Therefore, the dynamics of material choice in the model was implemented by means of an adjusted version of a Polya Urn Model (PUM), an iterative random sampling with over-replacement [14]. The PUM and its extensions are established methods for simulating path-dependent and self-reinforcing dynamics (e.g. contagion phenomena [15], technology adoption in economy [16] and evolution of languages [17]).

An ABM is the method of choice when a dynamic process that leads to the formation of an emergent pattern is to be investigated [18]. This task is different from classification and regression problems, where methods such as random forests may be used to analyze (large) datasets for correlations between certain outcomes, on the one hand, and certain features of the data, on the other hand. The difference is that in the first case the pattern is not known in advance whereas in the latter case the possible outcomes are known and the features are given, even if the dataset changes over time.

ABMs are often used to analyze various concepts in science because they account for the connection between autonomous actions of individual scientists and the overall scientific progress [19]. For example, Gilbert (1997) simulated the growth of scientific publications and distribution of citations per publication as the collective phenomena using the publications themselves as agents [20], Weisberg and Muldoon (2009) investigated the collective ability of a research community to discover scientific knowledge [21], and Balietti et al. (2015) investigated the correlation between disciplinary fragmentation and scientific progress as emerges from the social and epistemic interactions among agents representing scientists [22]. Similarly, the focus of the current work is on the diversity of the studied materials and its trend over time as a collective property of the DOM-PM research field. However, compared to the above-mentioned ABMs of science, the model is calibrated by empirical data from this research field and, therefore, based on fewer general assumptions.

## Materials and methods

### Model description

**General**. The model was written in the Python programming language, version 3.5 (Python Software Foundation, https://www.python.org/), data processing and statistical analysis was done in R [23]. The code and input data are freely available on https://github.com/nicolesanikast/ABM_science. The model description follows the ODD (Overview, Design Concepts, Details) protocol [24, 25]. Here we present the main modeling concepts; the full ODD description is available in section A of the S1 File.

#### Overview

**Purpose**. The model is an agent-based model aimed at investigating whether the FBMC dynamics can account for the observed imbalance in the choice of materials. To this end, the model simulates the evolution of this research field between 1990 and 2015 using the following processes: (i) formation of collaboration groups; (ii) exchange between agents within collaboration groups; and (iii) choice of materials for study by each collaboration group.

**Agents**. The model considers one type of agent that represents a researcher. Each agent is assigned a unique identity *i*, with *i* = 1, …, *N*, where *N* is the total number of agents. Each agent *i* is characterized by four state variables: (i) a tendency *t*_*i*_ that is selected uniformly at random from the interval [0, 1]; this variable reflects the tendency of the agent to choose DOM-PM combinations either based on their overall use in the past (when *t* is close to 1) or based on the use of either the DOM or PM component of these combinations (when *t* is close to 0); (ii) the duration of activity, in years, $d_{i}\in N$, with *d*_*i*_ ≤ *n*, where *n* is the number of simulated years. The duration is the number of years the given agent investigates the interaction between DOM and PM; (iii) a state *s*_*i*_ ∈ {active, inactive}. Only researchers with active state collaborate and carry out experiments. Inactive agents correspond to researchers that have switched to another research field or have retired. (iv) A list of collaborators, *c*_*i*_, that accumulates the identities of all other agents with whom agent *i* has collaborated in the simulated years. The four state variables were chosen such that the agents can make different choices regarding their research focus (choice of materials) and collaboration pattern and that the individual history of researchers in the field can be described. The number of state variables was kept low because this reflects the principle of parsimony in model design and makes it easier to link the aggregated behavior of the agents to concrete actions on the level of an individual agent, in particular a high or low preference for new material types (tendency *t*).

**Environment**. The environment in the model consists of two parts: (i) The sample space of all experimental settings. This is a hypothetical construct that consists of all possible combinations of DOM and PM available for studying. Each such combination is assigned a weight that corresponds to the number of times this combination was studied until (but not including) a given year. (ii) A control parameter, *C*. This parameter represents the overall preference of the scientific community to study DOM-PM combinations that were already studied in the past (*C* → 0), or to study new DOM-PM combinations, i.e., combinations where only the DOM or the PM constituent was studied in the past (*C* → 1). *C* defines to what extent the agents in the model apply frequency-based choice of material; low *C* means that many choices of materials are frequency based, high *C* means that FBMC is rare.

#### Process overview and scheduling

The model simulates the choice of DOM and PM between 1990 and 2015, where the simulated time is discrete and each time step represents a single year. The pseudo code for the main modeling steps is provided in section A.1 of the S1 File. In short, in each time step each active agent collaborates with three other active agents to form a collaboration group of four (this typical size of four of a collaboration group was derived from empirical data, see section A.3 of the S1 File). The formation of collaboration groups is either preferential, i.e., agents often collaborate with other agents with whom they have collaborated in previous time steps, or non-preferential, i.e., agents collaborate without a preference for those agents with whom they have already collaborated.

In each timestep, the members of each collaboration group update their tendencies (*t*) to equal the average tendency of the collaboration group ($\bar{t}$). Every collaboration group then compares its $\bar{t}$ to *C* and chooses three DOM-PM combinations to study (three is the empirical average of DOM-PM combinations studied per publication, see details in section A.3 of the S1 File). If $\bar{t}\geq C$, the DOM-PM combinations are picked randomly and proportionally to their use in previous time steps; if $\bar{t}<C$, they are picked randomly and proportionally to the use of either the DOM or the PM constituent in previous time steps:

$$\begin{array}{ccc} \text{P}{\left({\text{DOM}}_{y}-{\text{PM}}_{x}\right)}_{k,j} & \propto & \delta_{\left\{{\bar{t}}_{k,j}\geq C\right\}}\frac{W_{{\text{DOM}}_{y}}-{\text{PM}}_{x}}{n_{\text{exp},j-1}}\\ & & +\delta_{\left\{{\bar{t}}_{k,j}<C\right\}}\left\{ \begin{array}{cc} \frac{W_{{\text{DOM}}_{y}}}{n_{\text{exp},j-1}} & \text{if}\;b=1,b∼\text{Bernoulli}(0.5)\\ \frac{W_{{\text{PM}}_{x}}}{n_{\text{exp},j-1}} & \text{else,} \end{array}\right. \end{array}$$ (1)

where DOM_*y*_ and PM_*x*_ are some arbitrary DOM and PM types, ${\bar{t}}_{k,j}$ is the average tendency of agents in the *k*th collaboration group at the *j*th time step, *C* is the control parameter, *δ*_*a*_ = 1 if *a* is true and *δ*_*a*_ = 0 if *a* is false, *n*_exp,*j*−1_ is the number of experiments performed from 1990 until the (*j* − 1)th time step (*j* = 1990, …, 2015), *W*_*z*_ is the number of experiments performed with the material *z*, and *b* is a Bernoulli random variable with 50% probability of being 1. If, in the case of $\bar{t}<C$, by chance a DOM-PM combination is selected that was investigated before, this is counted as a repetition.

The counter showing how often each DOM-PM combination has been chosen is updated at the end of each time step (synchronous updating). Therefore, the choice of materials by each collaboration group is affected only by the choice of materials from previous time steps; this accounts for the situation where information regarding the experiments performed by others becomes known only once these experiments are published (here this is assumed to take a single time step, i.e., 1 year).

#### Design concepts

**Basic principles**. The model considers two approaches for the choice of materials: (i) when the average tendency of a collaboration group is above *C*, a given DOM-PM combination is selected based on the frequency at which it was studied in the past. The probability of choosing a given DOM-PM combination to study is proportional to its occurrence in the past, and therefore, whenever a given combination is studied the probability of studying it again increases. This process accounts for the observation that certain DOM-PM combinations are studied extensively while others are not [11]. (ii) When the average tendency is below *C*, a given DOM-PM combination is chosen with a probability that is proportional to the number of times either its DOM or its PM constituent was studied in the past. In this case, all DOM-PM combinations that share the same DOM (or PM) constituent have the same probability of being chosen. The more frequently this constituent was studied in the past, the more likely it is for any of the combinations containing it to be chosen again. In this situation, frequently studied DOM or PM are studied more often in subsequent steps with new counterparts. This process accounts for the observation that certain DOM and PM types have a large number of distinct material counterparts with which they have been studied [11].

The control parameter, *C*, is used to run the model under different conditions and the diversity of the DOM-PM combinations studied is the main characteristic of the experimental field analyzed here. It is expressed by the combination diversity index, (*D*_comb,*i*_ ∈ (0, 1]), which is the ratio between the unique number of DOM-PM combinations studied and the number of experiments performed up to and including the *i*th year (*i* = 1990, …, 2015) [11].

**Emergence**. For a given value of the parameter *C*, the model outputs several characteristics of the simulated research field: (i) a network that describes the pairwise occurrence of DOM and PM in the experiments during the simulated years (i.e., a simulated experimental network). In this network each node is either a DOM or a PM type and a link between a given pair of nodes means that the corresponding DOM and PM were studied together. The weight of a link corresponds to the number of times the given DOM-PM combination was studied during the simulated years; (ii) a time trend in *D*_comb_ over the simulated years; and (iii) a collaboration network among the agents (i.e., a simulated collaboration network). In this network each node is an agent and a link between two agents means that they collaborated (were part of the same collaboration group) at some time in the simulated years. The weight of a link is the number of times the two connected agents have collaborated. The total number of links in the collaboration network is the number of pairs of agents that have collaborated, and the sum of the link weights is the total number of all pairwise collaborations.

The simulated experimental network as well as the time trend in *D*_comb_ emerge from the collective actions of the autonomous agents. Unlike the simulated experimental network, the shape of the simulated collaboration network is predictable, as it is governed by the collaboration rules defined in the model (see section A.3 in the S1 File).

#### Details

**Initialization**. An array of all possible DOM-PM combinations (all possible combinations of DOM and PM types experimentally studied until and during 2015) is initialized with a weight of 1 for each combination. Because the combinations are later sampled proportionally to their weights, the initialization with 1 ensures that all combinations can be sampled. To account for the frequency of materials studied before 1990, the number of times each DOM-PM combination was studied before 1990 is added to the weight of that combination. We also ran the model with larger numbers of materials that are available for study and found that this does not change the behavior of the model; see details provided in section B of the S1 File. The simulation starts with a single collaboration group, which corresponds to the empirical number of new researchers entering the experimental field in 1990 (see S1 File for more details).

**Input data**. Input values are calculated from the empirical data for the years 1990–2015: (i) the overall number of PM and DOM types that were studied (94 PM types and 133 DOM types; see Sani-Kast et al. (2017) [11] for a description of how the PM and DOM types were defined); (ii) the number of new researchers in each year between 1990 and 2015; (iii) a vector of probabilities for duration of activity (in years). The vector lists the empirical probability that a given researcher, having been active for a given number of years, will continue to stay active for at least one more year. (iv) The average number of DOM-PM combinations studied in a single experimental publication; and (v) the average number of coauthors per publication (i.e., the average size of a collaboration group). See section A.3 in the S1 File for a list of all input values and explanatory figures.

### Model validation

#### Single simulation output

Simulation outputs were generated for different values of the control parameter *C*. For each such value, the simulation output was qualitatively compared to the empirical data: (i) the main structural features of the simulated and empirical experimental networks were visually compared; (ii) the magnitude and direction of the simulated and empirical time trends in *D*_comb_ were compared; and (iii) the segregation among the different collaboration groups as captured by the simulated collaboration network was compared (visually) to the one observed in the empirical collaboration network (i.e., co-authorship network). The latter was constructed from the co-authorship data of the experimental papers that studied the interaction between DOM and PM [11], here we only used the subset of papers that were published between 1990 and 2015 (248 publications in total, see Sani-Kast et al. (2017) [11] for details on the selection of the publications and the experiments reported in the publications). The bibliographic data of these publications was obtained from the Web of Science (https://apps.webofknowledge.com, accessed on 23.02.2017). By means of the VOSviewer software [26], the bibliographic data were converted, without further processing, to a co-authorship (i.e., collaboration) network.

#### Ensemble of simulated networks

Because of the inherent stochasticity in the simulation, both in the collaboration group formation and in the choice of materials (see section A.3 in the S1 File), simulation outputs differ in each model run. To account for this randomness, we performed 1000 model runs for different values of the control parameter *C*. Properties of the empirical collaboration network, empirical experimental network, and the empirical time trend in *D*_comb_ were compared to the distribution of these properties as obtained for the simulated collaboration networks, the simulated experimental networks, and the simulated time trends in the *D*_comb_ values, respectively.

The collaboration networks were compared in terms of: (i) the number of clusters: the number of distinct groups of agents (simulated networks) or researchers (empirical network) internally connected via collaboration ties but not connected to the rest of the network; (ii) the number of agents (or researchers) in the largest connected group; (iii) the average cluster size; and (iv) the degree assortativity, which is the correlation between the number of distinct collaborators for each pair of connected agents (or researchers).

The experimental networks were compared in terms of: (i) the average shortest path, where the shortest path between a given pair of nodes is the minimum number of links separating them; (ii) the diversity index (*D*_comb_); (iii) the degree assortativity, which is the correlation in the number of links between each pair of connected nodes. In the context of the experimental network, degree assortativity is the correlation between the number of unique DOM-PM combinations that were studied and contain a given DOM type and the number of unique DOM-PM combinations that were studied and contain a given PM type, for every pair of DOM and PM that were studied together. This approach makes it possible to use an extensive amount of information about the empirical experimental network in the validation of the model and, thereby, to make the validation robust, see S1 File for details.

## Results

### Segregated collaboration groups

**Fig 1:**
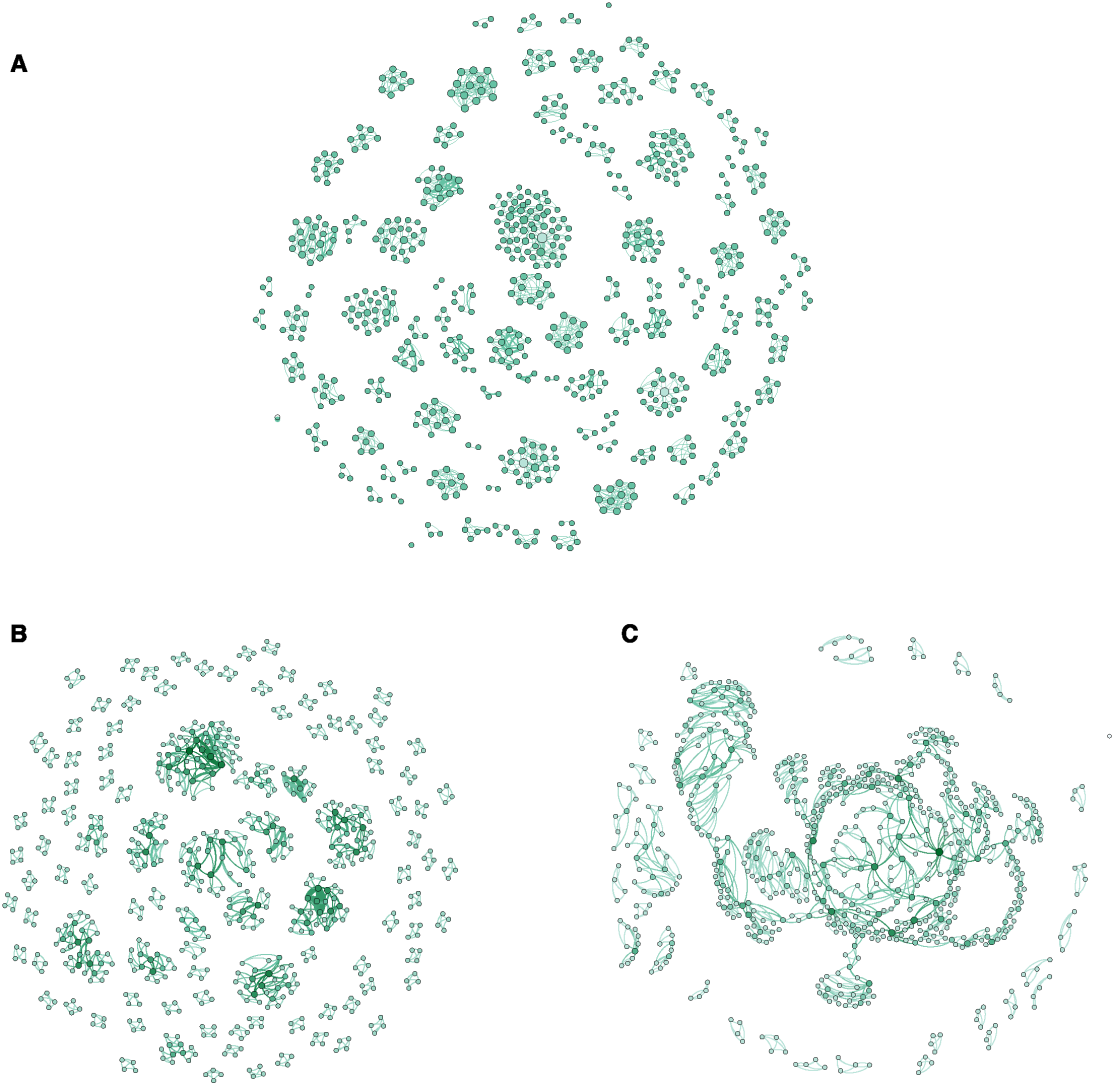
Collaboration networks. A: the empirical collaboration network among researchers who published experimental papers in 1990–2015 that study the interaction between DOM and PM. In the network, each node is a researcher, and a link between two researchers means that the two researchers have coauthored a publication at least once. B and C: simulated collaboration networks generated with the preferential and non-preferential collaboration approaches, respectively. In these networks, each node is an agent, and a link between two agents implies that the two were part of the same collaboration group at least once.

Fig 1A displays the empirical collaboration network, and Fig 1B and 1C display collaboration networks simulated with preferential collaboration and non-preferential collaboration approaches, respectively (see description below). In these collaboration networks each node is a researcher (Fig 1A, empirical network) or an agent (Fig 1B and 1C, simulated networks), and a link between two nodes means that the two researchers (agents) have collaborated at least once during 1990–2015.

In the *preferential* collaboration approach agents are assigned, when applicable, to collaboration groups with agents with whom they previously collaborated. This implies that newcomers collaborate with other newcomers (i.e., newcomers enter the research field already as part of collaboration groups with other newcomers), and agents already existing in the system prefer to maintain their already formed collaboration groups. The use of preferential collaboration as a model scenario is based on the observation that the empirical collaboration network is highly granulated with only few links between collaboration groups (Fig 1A). In the *non-preferential* collaboration approach, agents collaborate with other active agents without a preference for those with whom they have collaborated in the past.

The preferential collaboration approach results in a collaboration network that captures the main structural characteristics of the empirical collaboration network. Particularly, both the empirical and the collaboration network simulated with preferential collaboration are highly granulated, and at the same time contain some clusters of collaboration groups connected via a small number of nodes (i.e., researchers/agents that participate in several collaboration groups), compare Fig 1A and 1B. On the other hand, the non-preferential collaboration approach results in a completely different collaboration network, see Fig 1C. Specifically, it contains a single large connected component (i.e., many agents participating in overlapping collaboration groups) and fewer disconnected collaboration groups than in both the empirical collaboration network and the collaboration network simulated with preferential collaboration.

In addition to the qualitative similarities, a quantitative analysis (described in section C of the S1 File) supports the observation that the empirical collaboration network can result from a preferential collaboration approach similar to the one implemented in the model. Specifically, an ensemble of 1000 collaboration networks simulated with preferential collaboration and the empirical collaboration network all exhibit similar levels of granularity and connectedness among different clusters of collaboration groups. Specifically, the similarity was characterized by the number of disconnected clusters, size of the largest cluster, mean size of the clusters, and the correlation between the number of distinct collaborators for each pair of connected researchers/agents (i.e., degree assortativity). On the other hand, the distributions of these properties are substantially different for an ensemble of 1000 collaboration networks that were simulated using non-preferential collaboration (see Fig C in the S1 File).

Because the model assumes collaboration groups of a fixed size (i.e., the empirical average size of collaboration groups), it does not account for the existence of both large and small collaboration groups (see Fig B (a) in the S1 File). Consequently, the preferential collaboration approach captures the overall structure of the empirical collaboration network, but not the exact number of links in the collaboration network and the sum of link weights (see section C of the S1 File). Nonetheless, as shown before, the model does capture the segregation in the collaboration and the extent to which researchers mix among the different collaboration groups, a fact that will be utilized when the applicability of the model is discussed below.

### Frequency-based choice of materials

**Fig 2:**
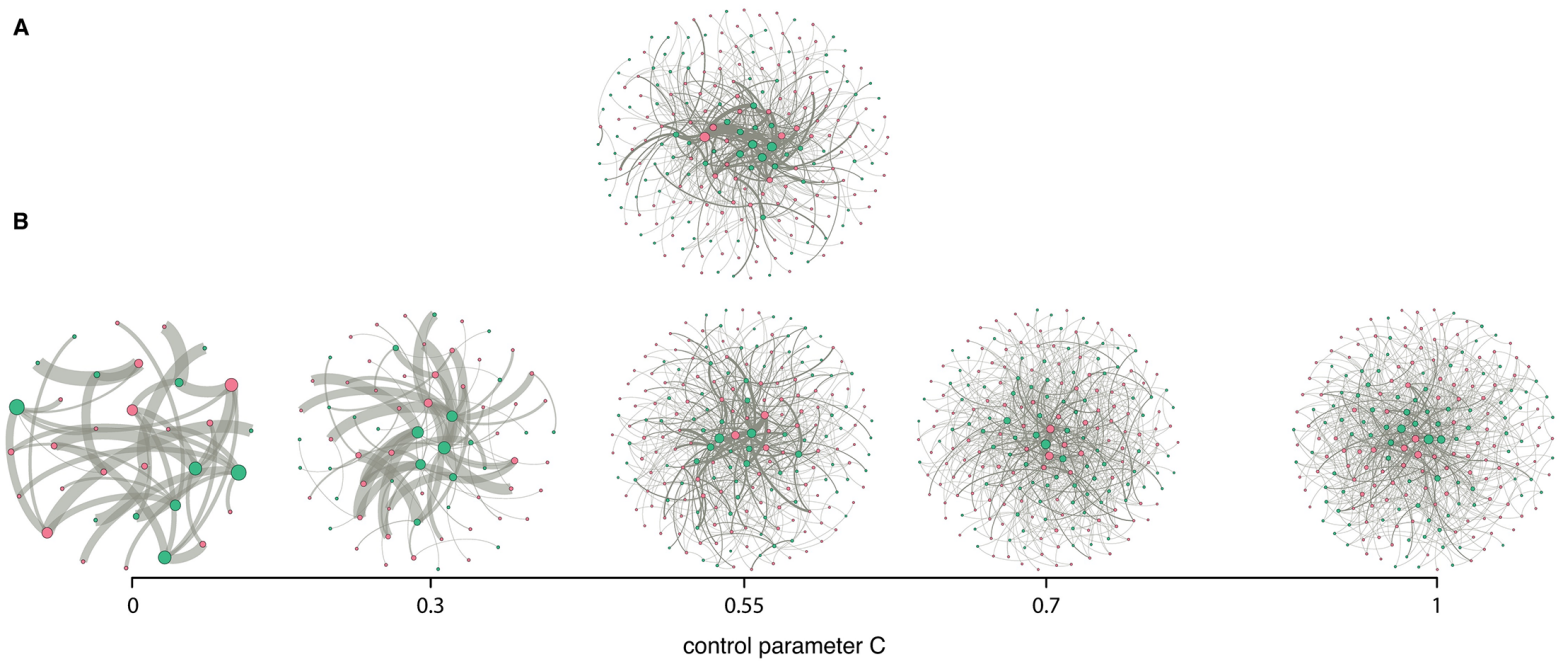
Experimental networks. Pink and green dots represent DOM and PM types, respectively. A link between two nodes means that the connected DOM and PM were studied together and the width of the link is proportional to the number of experiments studying the interaction between these DOM and PM types. A: The empirical experimental network. B: Simulated experimental networks obtained for different values of the control parameter *C*.

Fig 2A displays the empirical experimental network, and Fig 2B displays several simulated experimental networks generated for different values of the control parameter *C*. In each network, pink and green nodes correspond to DOM and PM types, respectively. Of the different simulated experimental networks, the experimental network obtained from simulations with *C* = 0.55 exhibits structural features that are most similar to the ones observed in the empirical experimental network. Particularly, it exhibits: (i) a dense core, in which nodes are highly connected to one another and have strong links. These nodes correspond to materials (either DOM or PM) and combinations of materials (DOM-PM) that were repeatedly chosen by the agents during the simulated research years. In the empirical network the dense core corresponds to DOM (PM) that were studied in many experiments with a wide range of material counterparts. (ii) Peripheral nodes that are connected to the central part of the network only via a small number of links. These nodes correspond to materials (either DOM or PM) that were chosen by the agents in the model only occasionally during the simulation. In the empirical network the peripheral nodes are materials that were studied infrequently and with a small number of material counterparts.

A detailed quantitative analysis further illustrates the performance of the model. The structure of the empirical experimental network was compared to ensembles of simulated experimental networks (1000 networks for each value of *C* from 0 to 1 in steps of 0.1 and also *C* = 0.55), using five descriptors: average shortest path, number of nodes, number of links, degree assortativity, and the diversity index (see subsection “Model validation” in the Methods section for a detailed explanation). This analysis shows in much detail that the experimental networks obtained with *C* = 0.55 have structural properties that are very similar to those of the empirical experimental network, see Fig E in the S1 File.

When *C* = 0.55, collaboration groups that have an average tendency, $\bar{t}$, above 0.55 are likely to choose DOM-PM combinations that were studied before, and therefore drive *D*_comb_ down. Collaboration groups with $\bar{t}$ below 0.55 choose DOM-PM combinations based on the frequency of usage of either the DOM or PM types and, therefore, they are more likely to choose DOM-PM combinations not studied before and subsequently increase *D*_comb_.

**Fig 3:**
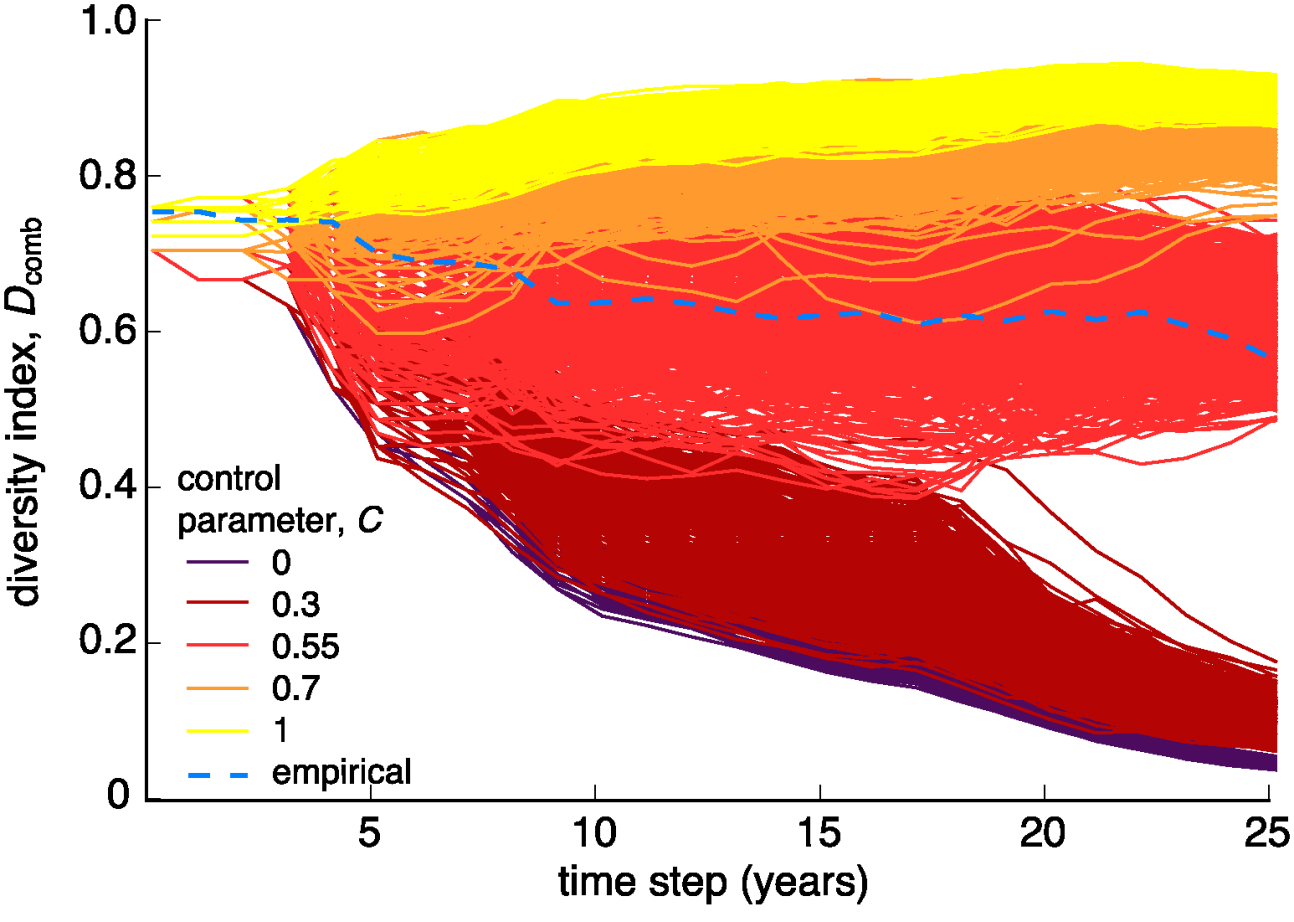
Trends in the diversity index, *D*_comb_. Empirical and simulated trends in *D*_comb_, which is the ratio between the number of unique DOM and PM combinations studied and the total number of experiments performed, between 1990 and 2015.

Fig 3 displays the time trend in *D*_comb_ between 1990 and 2015, for the empirical data, as well for the data obtained from 1000 model simulations for five values of *C*: 0, 0.3, 0.55, 0.7, and 1. Simulated experimental networks generated with a *C* value of 0.55 capture the magnitude and trend of the empirical *D*_comb_ closely. Similar to the empirical trend, the diversity of materials obtained for the simulated network with *C* of 0.55 is not monotonous. The fluctuations in *D*_comb_ reflect periods in which new DOM-PM combinations are chosen to be studied and periods where most chosen combinations were already studied before. However, for both the empirical and the model results for *C* = 0.55, *D*_comb_ slowly decreases over the years. In contrast, *D*_comb_ trends obtained from simulations using other *C* values systematically diverge from the empirical trend.

For *C* = 0.55, the choice of materials is more or less balanced between a choice of new DOM-PM combinations and a choice of DOM-PM combinations studied previously. However, even under these conditions the overall diversity of chosen DOM-PM combinations (i.e., *D*_comb_) decreases over time. This outcome originates from the definition of *D*_comb_ (for a detailed explanation, see section E of the S1 File). In short, from the derivation described in the S1 File, we observe that *D*_comb_ increases whenever the fraction of newly studied DOM-PM combinations in a given year, *i*, is larger than the *D*_comb_ value in the year *i* − 1. As explained before, when the control parameter *C* = 0.55, the fraction of newly studied combinations out of the experiments performed in any given year is about 0.5 (this is an approximate value because of the stochasticity of the model). *D*_comb_ primarily decreases because the simulation starts with a *D*_comb_ value of 0.75 (i.e., the empirical *D*_comb_ for the year 1989), which is greater than the fraction of newly studied combinations in each subsequent simulation step, namely ≈0.5 at *C* = 0.55.

## Discussion

### Choice of materials

The ABM presented here is able to reproduce, for *C* = 0.55, key characteristics of the research field investigated, including the final structure of the empirical experimental network and the empirical time trend in *D*_comb_. While the model (i) indicates how the empirical trend in material choice derives from the behavior of individual agents and (ii) shows how many choices made by the agents pick new vs. old DOM-PM combinations, the underlying causes for this trend remain to be studied. There are several possible reasons why researchers may prefer the study of certain materials over others so that the observed phenomenon of a frequency-based choice of materials occurs:

1. A material is used frequently because it has been used extensively in earlier experiments and the authors want to integrate their findings into the existing results for the same material. In other words, in this case it is mainly the “visibility” of a material that determines its further use.
2. A material is investigated frequently because it is highly hazardous (or considered highly hazardous), and this warrants repeated studies.
3. A material may not be particularly hazardous, but mechanistically interesting for other reasons, such as in the study of agglomeration or aging processes.
4. A material is useful (and established) as a general test system used to optimize measurement methods, i.e., here the focus is not on the materials, but on the development of measurement methods, and the materials are used extensively in order to provide a consistent basis for all measurements.

Regarding reason no. 2, it is unlikely that the observed decreasing diversity derives from a particular focus on materials that are highly hazardous, because it was recently recognized that much of the research into ENP risks focuses on materials that pose relatively low risk or whose risk is sufficiently well characterized [13].

Moreover, if the strong focus on certain materials that is present in the experiments was generally intended, then it is unclear why calls to expand the diversity of the materials are repeatedly being made [10, 12, 27].

Therefore, we think—in line with the reasoning of Maynard and Aitken (2016) [13]—that there is an imbalance in the choice of the DOM and PM types and that it is important to better understand the phenomenon of a frequency-based material choice, and the drivers that lead to this phenomenon. This question concerns the ability of the research community to direct (or redirect) the research focus. Once the underlying causes of the current dynamics become clearer, recommendations for future research can be made that take into account the self-reinforcing dynamics that, over time, can shift the research focus to potentially undesired directions.

Finally, the implications of the main simplifying assumptions made in the model are discussed briefly. In our model, each choice of a material increases its likelihood of being chosen again. This dynamics does not allow for a learning process in which research interests shift away from some materials that were studied extensively before. We made this assumption in the model because our earlier analysis of the empirical experimental network has shown that the most-studied materials (the ones in the core of the empirical network) were still studied in recent years [11]. In other words, there is no trend visible that would indicate a substantial shift of priorities in terms of materials studied.

Because the model was calibrated with the empirical data that describe the research field of DOM and PM interactions, the insights from the current modeling study cannot be simply generalized to other research fields. In order to investigate the presence of a similar dynamics in the design of experiments other than those studying the interaction between DOM and PM, the current model would have to be adjusted and calibrated using relevant empirical data.

### Effects of collaboration

The empirical collaboration network, see Fig 1A, is highly granulated, i.e., most researchers are members of small and isolated collaboration groups (the largest group includes 8.9% of the researchers). This is a feature that makes the DOM-PM research field different from many other fields. In other fields, often 80% or more of the researchers are connected to one another either directly or indirectly, which results in a large connected component comprising most nodes in the network [28, 29]. Such large connected components are found not only in coauthorship networks that span entire research areas such as biology, physics and mathematics [28], but also in coauthorship networks of very specific research areas (e.g. the publications regarding the Dengue disease by Brazilian authors published between 2001–2008 [30]).

There are several aspects that may explain the structural differences between the DOM-PM research field and other fields. A first observation is that many experiments on DOM-PM interactions do not require large teams and particularly expensive and logistically complicated equipment. Secondly, existing research teams may have “migrated” into this field from a wide variety of other disciplines, also driven by the increasing availability of funding for nanoparticle research. This is different from the progression of research and research collaborations in areas with a more continuous long-term development.

In our model, we compared two ways of collaboration between scientists in the field, preferential and non-preferential. Fig 1 shows that preferential collaboration reproduces the basic structure of the empirical collaboration network (Fig 1B), whereas non-preferential collaboration leads to an entirely different structure with (much) larger collaboration groups (Fig 1C).

In a separate analysis we investigated the effect of the collaboration approach on the diversity of the studied materials (details are provided in the S1 File). The results suggest that a change in the communication among researchers (e.g. moving from restrictive collaboration, represented here by preferential collaboration, to extensive collaboration, represented here as non-preferential collaboration) affects the experimental design and subsequently also changes the overall diversity of the studied materials (see section F of the S1 File). However, the effect is complex, since it exhibits non-linear dependency on multiple parameters (e.g. number of new agents entering the field, size of collaboration groups, and the control parameter *C*) and not very strong, see Fig G in the S1 File).

In light of these findings, we conclude that there is no direct relationship between the collaboration approach and the extent to which frequency-based choice of materials takes place. Before definite recommendations regarding collaboration approaches are made, rigorous investigation is needed into the current epistemic exchange among scientists in the research field of DOM-PM interactions. Regardless of the exact approach taken, it seems that a bottom-up approach to understanding and guiding scientific effort needs to consider the social structure of the research field in question. Our study supports the formerly stated realization that the understanding of the social interactions among scientists is imperative to the assessment of the scientific progress [31].

## Supporting information

S1 File Supporting information. This file includes the full model description according to the ODD protocol and additional analyses and figures.
(PDF)
