# Supplementary material for: The influence of past research on the design of experiments with dissolved organic matter and engineered nanoparticles
Source: PLoS One. 2018 May 7;13(5):e0196549. doi: 10.1371/journal.pone.0196549 (PMC5937778; doi:10.1371/journal.pone.0196549)
Supplement: S1 File — This file includes the full model description according to the ODD protocol and additional analyses and figures. (PDF) [file pone.0196549.s001.pdf]

Supporting Information to "The influence of past research on the design of experiments with dissolved organic matter and engineered nanoparticles"*

Nicole Sani-Kast,[1] Patrick Ollivier,[2] Danielle Slomberg,[3] Jérôme Labille,[3] Konrad Hungerbühler,[1] and Martin Scheringer[1,4,‡]

A Model description

A.1 Overview

General. The model was written in the Python programming language, version 3.5 (Python Software Foundation, https://www.python.org/), and the processing of the input data and statistical analysis was done in R [1]. The code and input data are freely available in https://github.com/nicolesanikast/ABM_science. The model description follows the ODD (Overview, Design Concepts, Details) protocol [2, 3].

Purpose. The model is an agent-based model (ABM) aimed at investigating whether the process of "frequency-based material choice" (FBMC) can account for the observed imbalance in the choice of materials. To this end, the model simulates the evolution of this research field between 1990 and 2015 using the following processes: (i) formation of collaboration groups; (ii) exchange between agents within collaboration groups; and (iii) choice of materials to study by each collaboration group.

Agents. The model considers one agent type that represents a researcher. Each agent is assigned a unique identity i, with $i = 1, \dots N$, where N is the total number of agents in the simulation. Each agent i is characterized by four state variables: (i) a tendency t_i that is chosen randomly from the interval $[0, 1]$. This variable reflects the tendency of the researcher to choose DOM-PM combinations to study either based on their overall usage in the past ($t \to 1$) or based on the usage of either the DOM or PM component of these combinations ($t \to 0$); (ii) duration of activity in years $d_i \in \mathbb{N}$, with $d_i \leq n$, where n is the number of simulated years. The duration is the number of years the given agent has been investigating the interaction between DOM and PM; (iii) a state $s_i \in \{\text{active}, \text{inactive}\}$. Only researchers with

*DOI: 10.1371/journal.pone.0196549
1 Institute for Chemical and Bioengineering, ETH Zürich, Zürich, Switzerland
2 Bureau de Recherches Géologiques et Minières (BRGM), Orléans, France
3 Aix-Marseille Université, CNRS, IRD, INRA, Coll France, CEREGE, Aix-en-Provence, France
4 RECETOX, Masaryk University, Brno, Czech Republic
‡ Corresponding author; E-mail: martin.scheringer@chem.ethz.ch

active state collaborate and carry out experiments. Inactive agents correspond to researchers that have switched to another research field or have retired; and (iv) a list of collaborators, c_i. It accumulates the identities of all other agents with whom agent i has collaborated during the simulated years.

Environment. The environment in the model consists of two parts: (i) the sample space of all experimental design settings. This is a hypothetical construct that consists of all possible combinations of DOM and PM available for studying. Each such combination is assigned a weight, which corresponds to the number of times this combination was studied until (but not including) a given year; and (ii) a control parameter, C. This parameter represents the overall preference of the scientific community to study DOM-PM combinations that were already studied in the past ($C \to 0$), or to study new DOM-PM combinations, where only the DOM or the PM constituent was studied in the past ($C \to 1$).

Collectives. In the model, researchers carry out experiments only as part of a collaboration group, i.e. a small group of researchers carrying out experiments and report the results together.

Scale. In the model, one time step simulates a single research year. The model runs for 26 time steps, corresponding to the research years between 1990 and 2015.

Process overview and scheduling. The model simulates the choice of DOM and PM between 1990 and 2015, where the simulated time is discrete and each time step represents a single year. The pseudo code for the main modeling steps is provided below. In short, in each time step each active agent collaborates with three other active agents to form a collaboration group of four (this size of the collaboration group is given by empirical data, see below, Input Data, on p. 5–6).

The members of each collaboration group update their tendencies (t) to match the average tendency of the collaboration group ($\bar{t}$). Every collaboration group then compares its $\bar{t}$ to C and chooses three DOM-PM combinations to study (i.e. the empirical average number of DOM-PM combinations studied per publication, see below, Input Data, on p. 5–6). The DOM-PM combinations are picked randomly and proportionally to their usage in previous time steps, if $\bar{t} \geq C$, or randomly and proportionally to the usage of either the DOM or the PM constituent in previous time steps, if $\bar{t} < C$:

$$
\begin{aligned}
\mathrm{P}(\mathrm{DOM}_y\text{-}\mathrm{PM}_x)_{k,j} \propto & \,\delta_{\{\bar{t}_{k,j} \geq C\}} \frac{W_{\mathrm{DOM}_y\text{-}\mathrm{PM}_x}}{n_{\exp,j-1}} \\
& + \delta_{\{\bar{t}_{k,j} < C\}} \begin{cases} \frac{W_{\mathrm{DOM}_y}}{n_{\exp,j-1}} & \text{if } b = 1, b \sim \text{Bernoulli}(0.5) \\ \frac{W_{\mathrm{PM}_x}}{n_{\exp,j-1}} & \text{else,} \end{cases}
\end{aligned} \qquad (1)
$$

where DOM_y and PM_x are some arbitrary DOM and PM types, $\bar{t}_{k,j}$ is the average tendency of agents in the kth collaboration group at the jth time step, C is the control parameter, $\delta_a = 1$ if a is true and $\delta_a = 0$ if a is false, $n_{\exp,j-1}$ is the number of experiments done from 1990 until the $(j-1)$th time step ($j = 1990, \ldots, 2015$), W_z is the number of experiments done with the material z, and b is a Bernoulli random

variable with 50% probability of being 1.

The counter showing how frequently each DOM-PM combination has been chosen is updated at the end of each time step (synchronous updating). Therefore, the choice of materials by each collaboration group is affected only by the choice of materials from previous time steps, accounting for the situation where information regarding the experiments done by others becomes publicly available only once the respective experiments are published (here it is assumed to take a single time step, i.e. 1 year).

Algorithm 1 Main model loop

Require: C, Mix {C is the control parameter, Mix is a logical variable, Mix = False corresponds to preferential collaboration}
  $researchers = N_1$ {number of researchers at the first time step}
  $experPrevious$ = InitExperiments()
  $groups$ = []
  for j in $1 \dots 26$ **do**
    $exper$ = copy($experPrevious$)
    APPEND Collaborate($researchers$, Mix) TO groups {create collaboration groups}
    for k in groups **do**
      $\bar{t} = \text{mean}(t_{group_k})$
      $t_{group_{k1}} \dots t_{group_{kn}} = \bar{t}$
      if $\bar{t} \geq C$ **then**
        $exper$[chooseMaterials1()] + 1 {choice of materials $\propto$ past usage}
      else
        $exper$[chooseMaterials2()] + 1 {choice of materials $\propto$ usage of either DOM or PM}
      end if
      if not DecideActive(k) **then**
        DELETE k FROM $groups$
        APPEND active($researchers_k$) TO $researchers$
      end if
    end for
    $experPrevious = exper$
    APPEND newResearchers[j] to $researchers$
end for

A.2 Design concepts

Basic principles. Sani-Kast et al. (2017) hypothesized that the choice of DOM-PM combinations to study is based on the frequency with which they were studied in the past, and therefore the experimental focus gradually concentrates on a subset of materials [4]. To inspect the plausibility of this hypothesis, we here model the choice of material using dynamics similar to a Polya urn model, a path-dependent and self-reinforcing random sampling with over-replacement [5].

The model considers two approaches for the choice of materials: (i) when the average tendency of a collaboration group is larger than C, a given DOM-PM combination is studied based on the frequency at which it was studied in the past. The probability of choosing a given DOM-PM combination to study is proportional to its occurrence in the past, and therefore, whenever a given combination is studied the probability of studying it again increases. This process accounts for the observation

that certain DOM-PM combinations are studied extensively while others are not [4]; and (ii) when the average tendency is smaller than C, a given DOM-PM combination is chosen with a probability that is proportional to the number of times either its DOM or its PM constituent was studied in the past. In this case, all DOM-PM combinations that share the same DOM (or PM) constituent have the same probability of being chosen. The more frequently this constituent was studied in the past, the more likely it is for any of the combinations containing it to be chosen again. In this situation, frequently studied DOM or PM are studied more often in subsequent steps with new counterparts. This process accounts for the observed phenomena of certain DOM and PM types having a large number of distinct material counterparts with which they have been studied [4].

The diversity of the studied DOM-PM combinations is the main characteristic of the experimental field analyzed here. It is expressed by the combination diversity index ($D_{\text{comb},i} \in (0, 1]$), which is the ratio between the unique number of DOM-PM combinations studied and the number of experiments carried out up to and including the ith year ($i = 1990, \ldots, 2015$) [4]. Because the control parameter C determines the probability of choosing new DOM-PM combinations to study, it is positively correlated with D_{comb}. The control parameter C is used here to generate simulation results with varying D_{comb} values.

Emergence. For a given value of the parameter C, the model outputs several characteristics of the simulated research field: (i) a network that describes the pairwise occurrence of DOM and PM in the experiments during the simulated years (i.e. a simulated experimental network). In this network each node is either a DOM or a PM type and a link between a given pair of nodes means that the corresponding DOM and PM were studied together. The weight of a link corresponds to the number of times the given DOM-PM combination was studied during the simulated years; (ii) a time trend in D_{comb} over the simulated years; and (iii) a collaboration network among the agents (i.e. a simulated collaboration network). In this network each node is an agent and a link between two agents means that they collaborated (were part of the same collaboration group) at some point during the simulated years. The weight of a link is the number of times the two connected agents have collaborated. The total number of links in the collaboration network is the number of unique pairs of agents that have collaborated, while the sum of the link weights is the total number of all pairwise collaborations.

The simulated experimental network as well as the time trend in D_{comb} emerge from the collective actions of the autonomous agents. Unlike the simulated experimental network, the shape of the simulated collaboration network is predictable, as it is governed by the collaboration rules defined in the model (see subsection "Details" below).

Adaptation. In each time step agents update their tendency (t) by adopting the average tendency of the group members with whom they currently collaborate. This process accounts for the transfer of information among collaborating researchers following, for example, scientific discussions.

Interaction. The agents directly interact with one another by forming collaboration groups and adapting their tendencies accordingly. Additionally, indirect in-

teraction among the agents is done via the choice of materials; as certain materials are being chosen by some agents, the future choice of these materials by other agents becomes increasingly more likely.

Stochasticity. The probability of choosing a certain DOM-PM combination is proportional to the frequency of its past usage or the usage of either its DOM or PM constituent. Due to the probabilistic nature of the material choice, the exact sequence of studied materials cannot be predicted. The stochastic element in the choice of materials accounts for other aspects of the experimental design that are not explicitly considered in the model, such as, for example, availability of materials, research questions tailored to a certain material, and limitations of experimental methods.

Collectives. Active agents from collaboration groups. These collaboration groups are identified by a unique number and are characterized by a list of agents comprising them. The collaboration groups are formed by the *Collaborate* function as described in the subsection "Sub models", below.

Observation. For each given C value, the model outputs: (i) the frequency of the chosen materials in a form of an experimental network; (ii) the evolution over time D_{comb}; and (iii) a collaboration network. The data are collected at the end of the simulated time steps.

A.3 Details

Initialization. An array of all possible DOM-PM combinations (all possible combinations of DOM and PM types studied until and during 2015) is initialized with a weight of 1 for each combination. Because the combinations are later sampled proportionally to their weights, the initialization with 1s ensures that all combinations can be sampled. To account for the frequency of studied materials before 1990, the number of times each DOM-PM combination was studied before 1990, is added to the weight of that combination. The simulation starts with a single collaboration group.

Input data. Input values are calculated from the empirical data for the years 1990–2015:

- The number of new researchers entering the field in each year between 1990 and 2015. For each year, $j = 1990, \ldots, 2015$, the number of new researchers is the number of authors first appear in the database in year j (i.e. did not author/coauthor any publication in the years 1990–$(j-1)$, Figure Aa.
- Empirical probabilities of activity duration. This is a vector of probabilities that describes for each duration activity (in years) $x = [1..25]$, the probability that a researcher, already active for x years, will stay active in the field for at least $x + 1$ years.
  The probabilities were calculated from the survival analysis of the empirical activity durations using a Kaplan-Meier curve [6]. For each one of the researchers authoring papers in the database, we calculated the duration of activity in the research field, i.e. the number of years between the first appearance of a given

author in any publication and the year of the his / her last appearance in any publication. Since for some researchers the last year of appearance is 2015, which is also the last year for which data was collected, we treated this cases as censured data, e.g. a researcher that first appear in 2013 and still publishes a paper in 2015 will have an activity duration of 3 years. However this data will be considered censored, which means that this researcher is not considered to have left the research field after 3 years. All researchers that last authored a publication at a year i, where $i < 2015$, are considered to have left the research field in the year i. Their activity durations are considered uncensored and are equal to the number of years between their first appearance and the year i. Using the Survival package [7] in R [1], a Kaplan-Meier curve was fitted to this censored data of the activity durations to obtain empirical survival probability (Figure Ab).

- The average number of authors per publication. Since the distribution of the number of coauthors per publication is skewed to the right (Figure Ba), the estimated mean was calculated with the "huberM" function in the package *robustbase* [8] in R [1], which is less sensitive to outliers. The mean of 3.8 was rounded up to 4 and served as the empirical collaboration group size in the model.

- The average number of experiments per publication, i.e. the average number of the unique DOM-PM combinations studied in the experimental papers in the database. Again, since the distribution of the number of unique DOM-PM combinations studied per publication is right-skewed (Figure Bb), we here used the "huberM" function to calculate the average number of experiments per publication (*robustbase* package [8] in R). The resulting mean of 3 (rounded up from 2.6) was used as an input to the model.

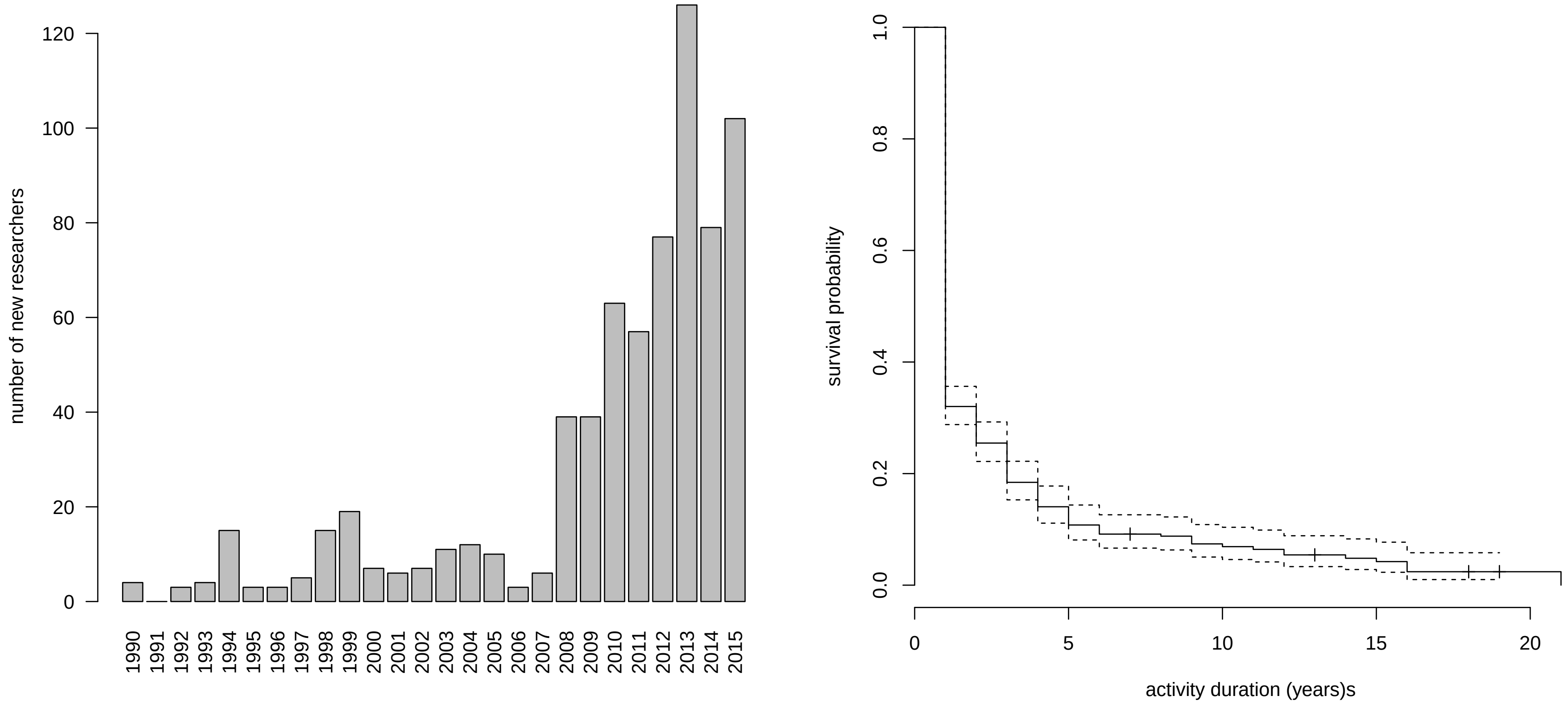

(a) (b)

Figure A: (a) The empirical number of new researchers entering the research field in the years between 1990 and 2015; (b) The Kaplan-Meier curve for the probability of duration activities of researchers between 1990 and 2015. The vertical axis is the probability of a researcher to continue his / her activity in the research field given he / she was active for the duration given on the horizontal axis. The '+' marks represent the censored data, those researchers that were still active in 2015 (the last year for which data was collected). The dashed line represent the 95% confidence interval of the probability to stay active for each duration activity.

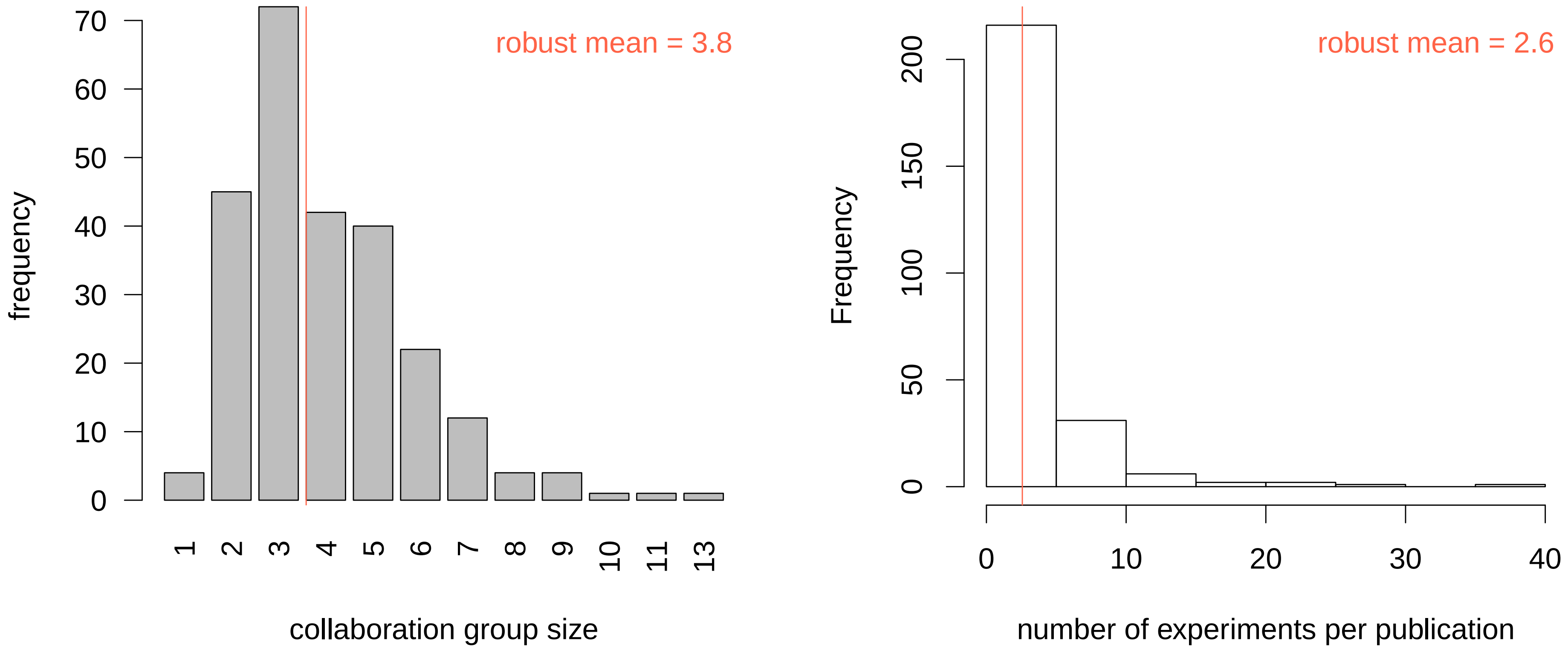

(a) (b)

Figure B: (a) The distribution and the robust mean of the number of coauthors per publication in the database. (b) The distribution and the robust mean of the number of unique DOM-PM combinations studied per publication in the database.

A.4 Submodels

InitExperiments. This function is executed once before the start of the simulations. It adds to the weight of each DOM-PM combination the number of times this combination was studied before 1990, as explained in the section "Initialization", above.

Collaborate. This function takes as an input a list of active agents that are not currently part of any collaboration group and assigns them to collaboration groups. As a default the argument "Mix" is set to *False*, meaning that collaboration groups are formed preferentially between researchers that have already collaborated in the past and that newcomers do not collaborate with existing agents (i.e. agents joining the system at time step j do not form collaboration groups with agents that have joined at time step $1 \cdots j-1$). Newcomers are assigned randomly to collaboration groups with other newcomers. When Mix is set to *True*, all agents that are free to collaborate (i.e. newcomers and active members of collaboration groups for which at least one member has turned inactive) are assigned randomly to collaboration groups.

DecideActive. This function takes as an input the identity of agents of a given collaboration group. Based on the activity-duration probability vector (see above section "Input data") and the duration of activity of each agent it decides whether or not this agent will stay active in the next time step: the probability that an agent will stay active after x years of activity is given by the xth entry in the activity-duration probability vector.

ChooseMaterials1 and ChooseMaterials2 For the choice of materials we differentiate between two cases: either (i) the average group tendency ($\bar{t}$) is larger than the control parameter C, or (ii) the average tendency is smaller than C.
If $\bar{t} \geq C$ the model executes the function chooseMaterials1: the choice of a given DOM-PM combination is proportional to the frequency of its usage in previous time steps. This dynamics results in an increase in the usage of certain DOM-PM combinations and decreases the overall D_{comb}. If $\bar{t} < C$ the model executes the function chooseMaterials2: the choice of a given DOM-PM combination is proportional to the frequency of usage of either its DOM or PM constituent in previous time steps. This results in an increase the number of DOM (PM) types studied with the given PM (DOM). It accounts for an increase in the overall D_{comb}.

The two above cases are expressed in equation 1 on p. 2 above.

B Effect of the number of available DOM and PM types

In the model, agents choose from 94 and 133 PM and DOM types, respectively, reflecting the number of the main DOM and PM types studied until and including 2015 [4]. It is important to note that the chemical identity of the materials is not a parameter that is needed by the model; the materials are characterized by the frequency with which the were chosen in the past. The number of materials from which the agents can choose is predefined.

In reality, however, researchers do not choose PM and DOM to study from a predefined and limited pool of materials. As new materials are constantly developed (e.g., PM coated with novel coating layers), the number of DOM and PM types available to study increases over the years, gradually opening new research opportunities.

Therefore, we investigate here what effect the predefined number of DOM and PM types has on the simulated results, in particular on the resulting D_{comb} value and the size of the simulated experimental network. To this end, the model was run with larger numbers of DOM and PM types from which agents can choose. Specifically, the model was run twice: first with 300 DOM types and 300 PM types, and then with 500 DOM types and 500 PM types. As the number DOM and PM types increases, the resulting number of possible DOM-PM combinations from which agents can choose increases quadratically (e.g. there are $300^2 = 90\,000$ and $500^2 = 250\,000$ possible DOM-PM combinations for 300 and 500 types of each DOM and PM, respectively). In these cases, the number of the DOM-PM combinations that are available to choose from exceeds by far the number of experiments actually performed during the simulated period (~ 1000). Accordingly, the agents in the model are no longer limited by the predefined number of DOM and PM types and, therefore, any repetition of DOM-PM combinations that may occur because of a limited number of materials to study is highly unlikely.

When the model was run with 500 DOM and 500 PM types (a 20-fold increase in the number of possible DOM-PM combinations compared to the original settings, where the numbers of PM and DOM types are 94 and 133, respectively), this resulted in a value of D_{comb} of 0.68 (compared to 0.64 for the original settings) and the experimental network grew twice in size. These results demonstrate that the gradual decrease in the D_{comb} values and the limited network size, as observed for the original settings, are highly unlikely to be caused by the predefined pool of DOM and PM types. Rather, the decrease in D_{comb} is likely due to the dynamics of the self-reinforcing (e.g. frequency-based) choice of materials.

C Collaboration networks

C.1 Principle component analysis

Analysis results

The empirical collaboration network was compared to an ensemble of 1000 collaboration networks that were simulated using preferential collaboration and 1000 collaboration networks simulated using non-preferential collaboration. Four descriptors were used for the comparison: (i) mean component size, MCS (i.e. the mean size of the connected components in the networks, where connected components are isolated groups of connected nodes), (ii) number of connected components, NC; (iii) size of the largest connected component, SLC; and (iv) the degree assortativity, DA (i.e. the correlation between the number of links for each pair of connected nodes). The comparison was done by performing a principle component analysis (PCA). In short, PCA is a statistical method that finds a set of orthogonal linear combinations (principle components, PCs) of descriptors that explain the largest proportion of variance in the data. Because the four descriptors substantially differ in their values (e.g. DA $= -1, \ldots, 1$ and NC $= 19, \ldots, 137$), the PCA was done on standardized

(or: normalized) data; the values of each descriptor were centered and scaled by subtracting the mean and dividing by the standard deviation of that descriptor (the mean descriptor values are MCS = 13, mean NC = 70, mean SLC = 260 and mean DA = 0.22). We denote the standardized values of the descriptors MCS, NC, SLC and DA as $\mathrm{MCS_{std}}$, $\mathrm{NC_{std}}$, $\mathrm{SLC_{std}}$ and $\mathrm{DA_{std}}$, respectively.

This analysis resulted in four PCs, with the first two PCs explaining 99% of the variability in the data (see subsection Code output, below):

- The first PC (PC1) contrasts the mean size of the connected components and the size of the largest connected component with the number of components and the degree assortativity:
  $0.5 \cdot (\mathrm{MCS_{std}} + \mathrm{SLC_{std}}) - 0.5 \cdot (\mathrm{NC_{std}} + \mathrm{DA_{std}})$.
  A network that scores a low PC1 value has a mean component size and a largest connected component that are smaller than the average mean component size and average largest component size, respectively. As well, in such a network the number of connected components and the degree assortativity are larger than the average values of these descriptors.

- The second PC (PC2) is a weighted average of the mean connected component size, the size of the largest connected component and the degree assortativity (with a low weight given to the number of connected components):
  $0.8 \cdot \mathrm{MCS_{std}} + 0.1 \cdot \mathrm{NC_{std}} + 0.6 \cdot \mathrm{DA_{std}}$.
  A network with a negative PC2 value has a mean component size that is smaller than the average mean component size and / or a degree assortativity that is smaller than the mean degree assortativity. A network with a positive PC2 value has a mean component size and / or a degree assortativity that are larger than the average values.

Figure C depicts the distribution of the simulated and the empirical collaboration networks in the lower dimensional space spanned by the first two PCs. The empirical collaboration network has negative values for both PC1 and PC2. These scores mean that the empirical collaboration network has many small size connected components and does not contain a very large connected component. All this combines to a description of a collaboration network with a large number of isolated collaboration groups. This description fits the empirical collaboration network as depicted in Fig.1a in the main text. The ensemble of the collaboration networks that were simulated with preferential collaboration are located in a small region that is in proximity to the empirical collaboration network (Figure C).

The collaboration networks simulated with non-preferential collaboration are spread over a large region in the two dimensional space spanned by PC1 and PC2 (Figure C). They span a large range of PC2 values and a rather small range of PC1 values. Their distribution implies that the collaboration networks generated with non-preferential collaboration mainly vary in their mean component size and their degree assortativity. However, these networks are similar to one another in a sense that they all have small numbers of components and have one connected component that is substantially larger than the others (high PC1 values).

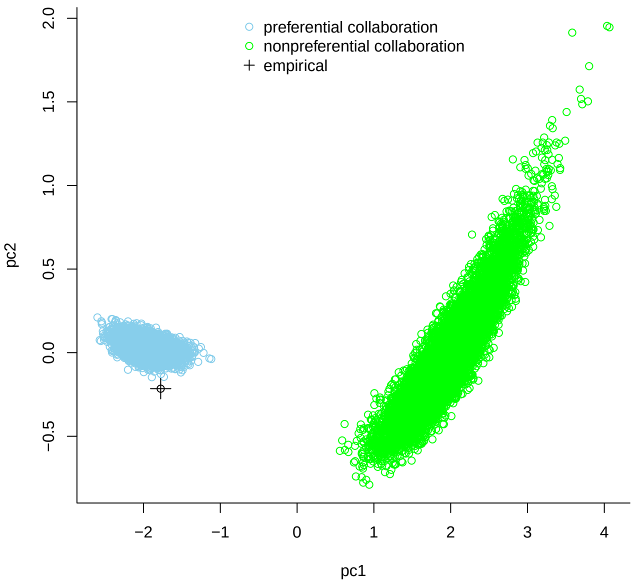

Figure C: The spatial distribution of the collaboration networks in the two dimensional space spanned by the first two principle components (PCs).

Code output

The following is the output of the PCA of the collaboration networks. The analysis was done using the *princomp* function in the statistical software R [1].

```
Importance of components:
                          Comp.1     Comp.2      Comp.3      Comp.4
Standard deviation      1.979677 0.22345272 0.147803026 0.095411183
Proportion of Variance 0.979780 0.01248278 0.005461434 0.002275823
Cumulative Proportion  0.979780 0.99226274 0.997724177 1.000000000

Loadings:
                      Comp.1 Comp.2 Comp.3 Comp.4
mean.component.size    0.496  0.808  0.219  0.230
n.components          -0.503  0.151 -0.266  0.809
size.giant.component   0.501        -0.860
degree.assortativity  -0.500  0.563 -0.377 -0.540
```

C.2 The effect of collaboration group size

Fig. D depicts the effect of the collaboration group size on the number of links and the total sum of link weights in the simulated collaboration networks. The number of links corresponds to the number of collaborations between pairs of distinct agents (simulated networks) / researchers (empirical network). The sum of link weights corresponds to the total number of collaboration ties made during the analyzed years (including repeated collaborations between the same individuals).

In a collaboration group of size four, each agent is connected to other three agents resulting in the creation of 6 collaboration ties (number of unique pairs of agents

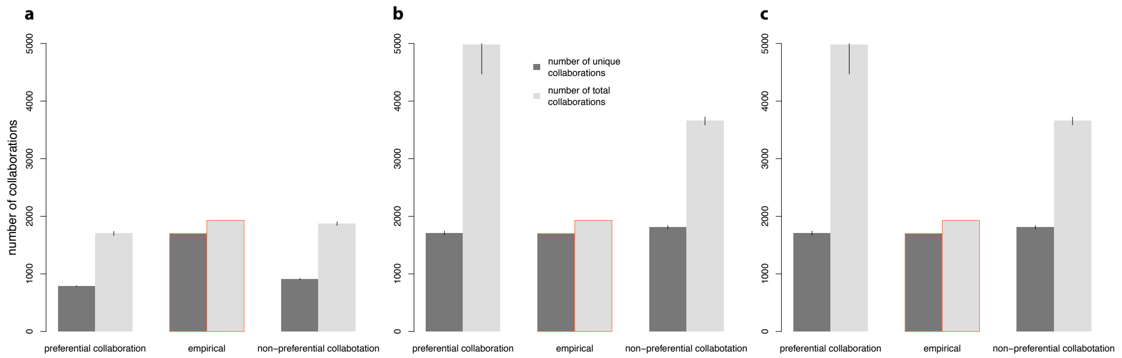

Figure D: A comparison in the number of distinct collaborations (i.e. number of links) and the number of total collaborations (i.e. sum of link weights) between the empirical collaboration network and simulated collaboration networks generated with collaboration groups of size three (a), four (b) and five (c). For each collaboration group size and for each collaboration approach (i.e. preferential and non-preferential collaboration), 1000 networks were simulated. The heights of the bars are the mean values in each ensemble of 1000 networks and the error bars are the mean plus minus one standard deviation.

in a group of four agents) between the agents. In a collaboration group of size five, 10 collaboration ties are created between members of the group. Finally, in a collaboration group of size three, only 3 collaboration ties are created between the collaborating agents. Therefore, as the size of the collaboration groups increases the total number of collaboration ties and the number of collaboration ties between distinct pairs of agents increases as well.

This effect is evident in Fig. D: (i) when collaboration groups were simulated to have three agents, the model underestimated the number of links in the collaboration network compared to the empirical network, more than it did when collaboration group size was set to four (compare Fig. Da to Fig. Db); and (ii) and when the collaboration group size was set to five agents, the overestimation of the overall number of collaborations taken place increases compared to simulations with collaboration group of size four (compare Fig. Dc to Fig. Db).

D Experimental networks

D.1 Principle component analysis

Analysis results

The empirical network was compared to an ensemble of simulated experimental networks, where the ensemble comprises 1000 experimental networks for each value of the control parameter $C \in [0, \ldots, 1]$. The networks were compared using five descriptors: (i) average path length (AVP), where path length between a given pair of nodes is the minimal number of links separating them. The average path length is taken over the paths between all node pairs in the network; (ii) number of nodes in the network (NN); (iii) number of links (NL); (iv) degree assortativity (DA), which is the correlation between the number of links of each pair of connected nodes; and (v) the diversity of the studied DOM-PM combinations, D_{comb}.

Similar to the collaboration networks, the comparison of the experimental net-

works was done using PCA. Here, too, the data was standardized (the values of each descriptor were transformed by subtracting the mean and dividing by the standard deviation) prior the analysis (the mean descriptor values are: mean AVP = 3.5, mean NN = 148, mean NL = 393, mean DA = -0.29, mean D_{comb} = 0.49). We denote the standardized values of AVP, NN, NL, DA and D_{comb} as AVP_{std}, NN_{std}, NL_{std}, DA_{std} and $D_{\text{comb}_{\text{std}}}$, respectively.

The resulting five PCs and their weights are listed in the "Code output" section, below. The first two PCs explain 98% of the variability in the database:

- PC1 contrasts the average path length with the rest of the descriptors:
  $0.4\text{AVP}_{\text{std}} - 0.4(\text{NN}_{\text{std}} + \text{NL}_{\text{std}} + \text{DA}_{\text{std}} + D_{\text{comb}_{\text{std}}})$.
  A network that has a low PC1 value has a short average path length and large value for the rest of the descriptors. Since the degree assortativity $\in [-1, 1]$ has a negative coefficient in PC1, a low PC1 value means that the degree assortativity becomes less negative.

- The second PC (PC2) contrasts the average path length and the degree assortativity with the number of nodes:
  $0.5\text{AVP}_{\text{std}} + 0.8\text{DA}_{\text{std}} - 0.1\text{NN}_{\text{std}}$.
  A network with low PC2 value has a degree assortativity that is smaller than the average degree assortativity (i.e. negative degree assortativity), a relatively short average path length and a large number of nodes.

The distribution of the experimental networks in the lower dimensional space spanned by the first two PCs is depicted in Figure E. There is a clear clustering of the experimental networks in the PC1–PC2 space according to the control parameter C used in the simulations. Particularly, networks generated with small C values have positive PC1 values and networks generated with large C values have negative PC1 values. In terms of PC2 all networks are scattered around PC2 = 0, however, as C gets larger, the scatter in the networks around PC2 = 0 gets smaller, therefore the corresponding networks become more similar to one another in terms of their PC2 values. This observation implies that, as the diversity of the studied DOM-PM combinations increases (C gets larger), the networks become more similar to one another in terms of the average path length, the number of nodes and the degree assortativity.

The empirical experimental network has both a negative PC1 value and a negative PC2 value. It is positioned in the region occupied by experimental networks simulated with $C = 0.55$, black cross in Figure E; accordingly, the structural features of the empirical experimental network are similar to the features of these simulated networks.

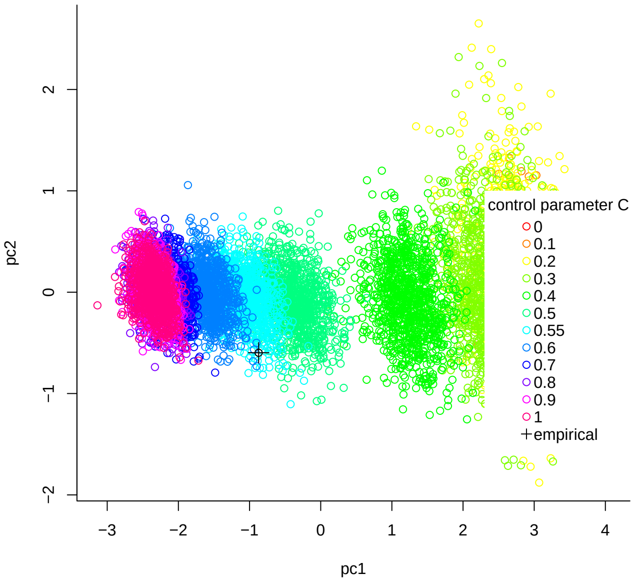

Figure E: The distribution of the simulated experimental networks ("o") and the empirical experimental network ("+") in a lower dimensional space spanned by the first two principle components. The points are colored according to the value of the control parameter C used in the simulation, as indicated in the legend.

Code output

The following is the output of the principle component analysis of the experimental networks. The analysis was done using the *princomp* function in the statistical software R [1].

```
Importance of components:
                          Comp.1     Comp.2     Comp.3      Comp.4
Standard deviation     2.1822651 0.34980544 0.25389671 0.213195707
Proportion of Variance 0.9524562 0.02447277 0.01289271 0.009090482
Cumulative Proportion  0.9524562 0.97692893 0.98982164 0.998912117

Comp.5
0.073752392
0.001087883
1.000000000

Loadings:
                     Comp.1 Comp.2 Comp.3 Comp.4 Comp.5
average path          0.444  0.545 -0.363  0.611
n.vertices           -0.448 -0.149  0.477  0.740
n.links              -0.453        -0.546        -0.701
degree assortativity -0.437  0.821  0.254 -0.264
diversity            -0.453        -0.527         0.712
```

E The relationship between the control parameter C and $D_{\mathbf{comb}}$

The control parameter C governs the fraction of newly studied DOM-PM combinations in each time step. For example, when the control parameter $C = 0.5$ there is at least a 50% probability that a given collaboration group will choose DOM-PM combinations that were studied in the past. Since all groups choose materials independently from one another, when the control parameter $C = 0.5$ the fraction of newly studied DOM-PM in a given time step is about 0.5.

The following inspects the relationship between the control parameter C and the change in D_{comb}.

We denote x_i and y_i as the number of unique DOM-PM combinations studied and the number of experiments done until and during the ith time step, respectively. Then, $D_{\text{comb},i}$ is given by

$$\frac{x_i}{y_i} \tag{2}$$

The number of new DOM-PM combinations studied and the number of experiments done in time step $i+1$ are denoted by n_{i+1} and e_{i_+1}. Then $D_{\text{comb},i+1}$ is given by

$$\frac{x_i + n_{i+1}}{y_i + e_{i_+1}}. \tag{3}$$

A decrease in D_{comb} occurs whenever $D_{\text{comb},i+1} < D_{\text{comb},i}$, that is when

$$\frac{x_i + n_{i+1}}{y_i + e_{i_+1}} - \frac{x_i}{y_i} < 0. \tag{4}$$

Using algebraic manipulation and rearrangement we observe that D_{comb} decreases whenever

$$\frac{n_{i+1}}{e_{i_+1}} < \frac{x_i}{y_i}. \tag{5}$$

Therefore, D_{comb} decreases when the fraction of newly studied DOM-PM combinations in a given year is less than D_{comb} of the previous year. When the control parameter $C = 0.55$, $\frac{n}{e} \approx 0.55$ in each simulation step, consequently, as long as $D_{\text{comb}} > 0.55$, the trend in D_{comb} is a decreasing trend.

F Effect of collaboration approach on $D_{\mathbf{comb}}$

We compared the effect of the collaboration approach (i.e. preferential vs. non-preferential) on D_{comb}. For different C values (0, 0.3, 0.4, 0.55, 0.6, 0.7, 1), we subtracted the average diversity of DOM-PM combinations from 100 model simulations obtained using preferential collaboration from the average values obtained from 100 model simulations using non-preferential collaboration. For each C value 50 such differences were calculated. The results are shown in Figure Ga.

We observe that non-preferential collaboration has a non-trivial effect on D_{comb}. This effect is an emergent phenomenon of the simulated system as it exhibits non-linear dependency on multiple parameters (i.e. the value of the control parameter

C, the number of agents entering the field and the size of the collaboration group). Particularly, the effect of non-preferential collaboration on D_{comb} has two regimes: (i) when the number of agents is small (before time step 19, which corresponds to 2009, see Figure Aa), non-preferential collaboration enhances the effect of C (increases D_{comb} when C is high and reduces it when C is low); (ii) when the number of agents is high (after time step 19, which corresponds to the year 2009, see Figure Aa), non-preferential collaboration reduces D_{comb} for high C values and increases D_{comb} for low C values.

Non-preferential collaboration incorporates newcomers into established collaboration groups and increases the rate at which non-newcomers change their collaboration groups. As a result of the fast mixing, there are fewer groups with extreme tendencies compared to when collaboration is preferential, see Figure F. Depending on the value of C, the change from preferential to non-preferential collaboration affects D_{comb}: when $C < 0.5$ the majority of collaboration groups have mean tendency above C and the main approach to choosing materials is governed by the frequency at which DOM-PM combinations were studied. Therefore, when $C < 0.5$, non-preferential collaboration reduces D_{comb} compared to preferential collaboration. When $C > 0.5$, the majority of collaboration groups have a mean tendency that is lower than C and choose materials to study based on the frequency of either DOM or PM types, which eventually increases D_{comb}. However, this effect is reversed at time step 19, when the number of new agents that enter the field substantially increases (Figure Aa).

This may be due to the complex effect of non-preferential collaboration on the distribution of average group tendencies: on the one hand, non-preferential collaboration reduces the occurrence of collaboration groups with extreme (either high or low) average tendencies, on the other hand, it increases the occurrence of collaboration groups with mid-value average tendencies (Figure F). Because of this dual effect the final outcome is complex and can be analyzed mainly via simulations.

A change in the collaboration approach leads to a change of up to 0.05 units in D_{comb}, which is small: for comparison, the decrease in the empirical D_{comb} is 0.19 units (a decrease from a diversity index of 0.75 in 1990 to 0.56 in 2015).

To verify that this effect is not due to the inherent stochasticity in the model, we analyzed the change in diversity between different model runs using only preferential collaboration. The diversity did change between different model runs, however, no systematic change was observed in D_{comb} when different values of C were employed. (Figure Gb). Therefore, we conclude that the effect of the collaboration approach is systematic and is not due to random effects in the model.

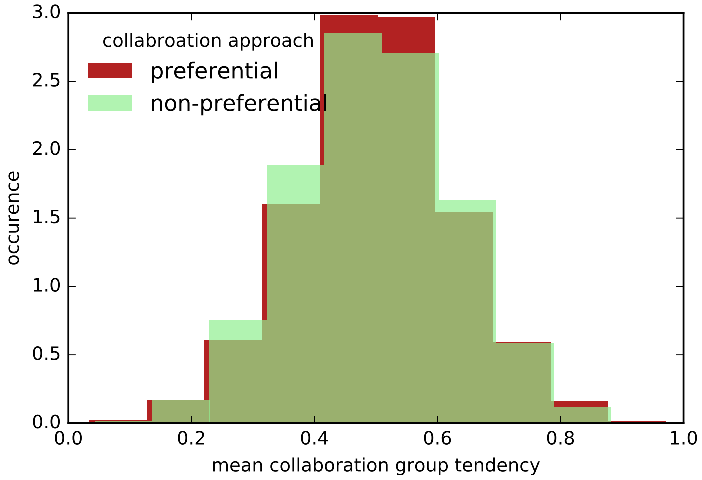

Figure F: The effect of the collaboration approach on the distribution of mean tendencies of the collaboration groups. For each collaboration approach (preferential and non-preferential), 1000 model simulations were carried out, the distributions depicted are aggregated over all 1000 simulation outputs.

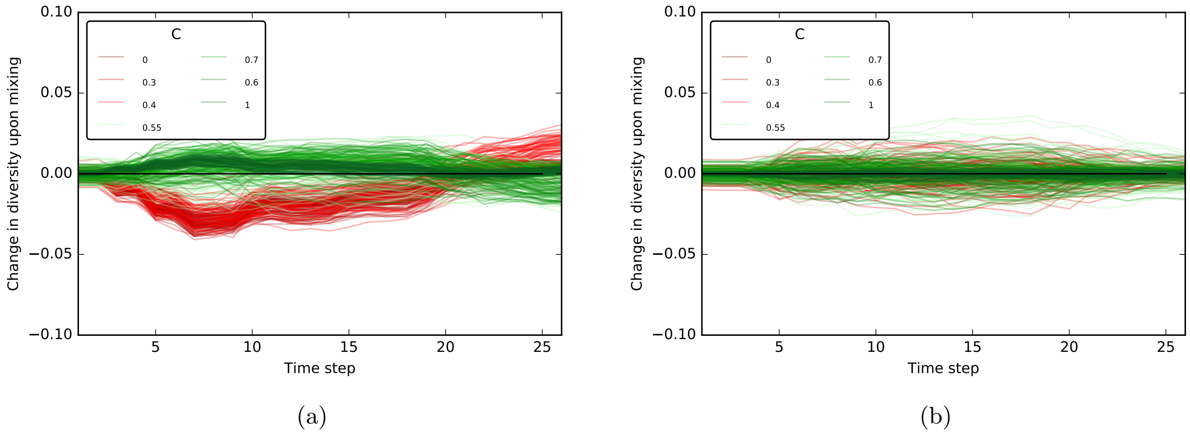

Figure G: (a) The difference in the diversity of the studied DOM-PM combinations obtained from simulations with non-preferential collaboration and simulations with preferential collaboration. Each line represents the difference in D_{comb} between the mean diversity values obtained from 100 simulations using non-preferential collaboration and the mean diversity values obtained from 100 simulations using preferential collaboration. For each value of the control parameter C (as detailed in the legend), 50 such comparisons were made. (b) The same analysis as before, only here each line is the difference in mean D_{comb} values obtained between two ensembles of simulations outputs (100 model outputs each) only using the preferential collaboration approach.

References

[1] R Core Team. R: A Language and Environment for Statistical Computing; 2013. Available from: http://www.r-project.org/.

[2] Grimm V, Berger U, Bastiansen F, Eliassen S, Ginot V, Giske J, et al. A standard protocol for describing individual-based and agent-based models. Ecological

Modelling. 2006;198(1):115–126. doi:10.1016/j.ecolmodel.2006.04.023.

[3] Grimm V, Berger U, DeAngelis DL, Polhill JG, Giske J, Railsback SF. The ODD protocol: a review and first update. Ecological Modelling. 2010;221(23):2760–2768. doi:10.1016/j.ecolmodel.2010.08.019.

[4] Sani-Kast N, Labille J, Ollivier P, Slomberg D, Hungerbühler K, Scheringer M. A network perspective reveals decreasing material diversity in studies on nanoparticle interactions with dissolved organic matter. Proceedings of the National Academy of Sciences. 2017;114(10):E1756–E1765. doi:10.1073/pnas.1608106114.

[5] Wei LJ, Wei, J L. Polya's Urn Model. In: Wiley StatsRef: Statistics Reference Online. Chichester, UK: John Wiley & Sons, Ltd; 2014.Available from: http://doi.wiley.com/10.1002/9781118445112.stat05795.

[6] Rich JT, Neely JG, Paniello RC, Voelker CCJ, Nussenbaum B, Wang EW. A practical guide to understanding Kaplan-Meier curves. Otolaryngology–head and neck surgery : official journal of American Academy of Otolaryngology-Head and Neck Surgery. 2010;143(3):331–6. doi:10.1016/j.otohns.2010.05.007.

[7] Therneau TM, Grambsch PM. Modeling survival data: extending the Cox Model. Statistics for Biology and Health. New York: Springer; 2000. Available from: http://link.springer.com/10.1007/978-1-4757-3294-8.

[8] Maechler M, Rousseeuw P, Croux C, Todorov V, Ruckstuhl A, Salibian-Barrera M, et al.. Robustbase: basic robust statistics R package; 2016. Available from: http://cran.r-project.org/package=robustbase.
